# Local Corticosteroid Injections versus Surgical Carpal Tunnel Release for Carpal Tunnel Syndrome: Systematic Review and Meta-Analysis

**DOI:** 10.3390/life12040533

**Published:** 2022-04-04

**Authors:** Luise Schäfer, Nicola Maffulli, Alice Baroncini, Jörg Eschweiler, Frank Hildebrand, Filippo Migliorini

**Affiliations:** 1 Department of Orthopaedic, Trauma, and Reconstructive Surgery, RWTH University Hospital, 52074 Aachen, Germany; luiseschaefer83@gmail.com (L.S.); alice.baroncini@gmail.com (A.B.); joeschweiler@ukaachen.de (J.E.); fhildebrand@ukaachen.de (F.H.); 2 Department of Medicine, Surgery and Dentistry, University of Salerno, 84081 Baronissi, Italy; n.maffulli@qmul.ac.uk; 3 School of Pharmacy and Bioengineering, Faculty of Medicine, Keele University, Stoke on Trent ST4 7QB, UK; 4 Centre for Sports and Exercise Medicine, Barts and the London School of Medicine and Dentistry, Mile End Hospital, Queen Mary University of London, London E1 4DG, UK

**Keywords:** carpal tunnel syndrome, steroid injections, carpal tunnel release

## Abstract

Introduction: Carpal tunnel syndrome (CTS) is the most common entrapment neuropathy. This meta-analysis compared local steroid injections (LSIs) versus carpal tunnel release (CTR) for the management of CTS. Neurophysiological parameters, patient-reported outcome measures (PROMs), and the complication rate were investigated. We hypothesized that LSIs may represent an effective and safe alternative to surgical management. Methods: This systematic review was conducted according to the 2020 PRISMA statement. All the clinical investigations comparing LSIs versus CTR for carpal tunnel syndrome were accessed. In March 2022, the following databases were accessed: Pubmed, Web of Science, Google Scholar, and Embase. No time constrains were used for the search. The risk of bias and statistical analyses were conducted using the Review Manager Software 5.3 (The Nordic Cochrane Collaboration, Copenhagen). Results: Data from 1096 procedures were retrieved. The mean follow-up was 12.3 (1 to 58) months. The mean age of the patients was 51.1 ± 4.6. Nocturnal paraesthesia (*p* < 0.0001) and visual analogue scale (*p* < 0.0001) were greater in the LSIs cohort. No difference was found in the functional (*p* = 0.2) and symptom (*p* = 0.4) subscales of the Boston Carpal Tunnel Questionnaire (BCTQ), median nerve distal motor latency (*p* = 0.9), median nerve motor amplitude (*p* = 0.7), median nerve sensory conduction velocity (*p* = 0.4), or median nerve sensory amplitude (*p* = 0.3). No difference was found in terms of minor complications (*p* = 0.9). No major complications were observed within the duration of follow-up. Conclusion: Both CTR and LSIs were effective and feasible in reducing symptoms of carpal tunnel syndrome. Though LSIs led to greater pain relief, this superiority was not permanent. Irrespective of the severity of the symptoms, current evidence suggests that a cycle of LSIs may be considered in patients with CTS. However, patients must be aware that LSIs may not be the definitive therapy, and CTR should be recommended.

## 1. Introduction

Carpal tunnel syndrome (CTS) is the most common entrapment neuropathy [1,2]. CTS is caused by localized mechanical compression and consequent local ischemia of the median nerve in the carpal tunnel [3,4]. In the general population, the prevalence of CTS may also depend on the diagnostic criteria used. Clinical diagnostic criteria overestimated the prevalence of CTS when compared to electrophysiology. When clinical and electrophysiological diagnostic criteria have been considered simultaneously, the prevalence is 2.7% [1,5]. The incidence of CTS is rising [6]. Women are three times more commonly affected than men of the same age [1,7]. Obesity, diabetes mellitus, thyroid disorders, rheumatoid arthritis, previous wrist factures, and pregnancy are well-established risk factors for CTS [5,8,9]. Repetitive flexion and extension of the hand and wrist have also been associated with the occurrence of CTS [6,10,11]. Numbness, paresthesia, and pain, especially at night, are the most common symptoms of CTS. If left untreated, patients with CTS may develop weakness and atrophy of the abductor and opponens pollicis muscle complex [6,12]. Local steroid injections (LSIs) and surgical carpal tunnel release (CTR) are commonly performed in patients with CTS [13,14,15,16,17]. However, the optimal modality is still unclear, and no consensus on the best approach has been reached [17,18]. To the best of our knowledge, systematic reviews or meta-analyses comparing LSIs versus CTR are missing. In 2018, Klokkari et al. performed a systematic review comparing the efficacy of conservative versus surgical management for CTS [19]. Since their study, more recent clinical investigations comparing LSIs versus CTR have been published [20,21,22,23].

This meta-analysis compared LSIs versus CTR in terms of symptoms and function in patients with CTS. Neurophysiological parameters and the time to return to normal activities were also considered. We hypothesized that LSIs may represent an efficacious and safe alternative to surgical management.

## 2. Materials and Methods

### 2.1. Eligibility Criteria

All clinical investigations comparing LSIs versus CTR for carpal tunnel syndrome were accessed. In accordance with the authors’ language capabilities, articles in English, German, Italian, French, and Spanish were eligible. Only level I studies, in accordance with the Oxford Centre of Evidence-Based Medicine [24], were considered. For studies investigating LSIs, all types of steroids were included (e.g., betamethasone and prednisolone). Studies which combined LSIs with local anesthetics (e.g., lidocaine) were included. Injections with other compounds such as hyaluronic acid, platelet-rich plasma, dextrose 5%, or lidocaine alone were not eligible. Studies which combined the treatment (LSIs/CTR) with other conservative procedures were excluded, as were those that applied experimental physiotherapy or orthosis. Abstracts, posters, comments, reviews, and editorials were not considered; nor were animal, in vitro, biomechanical, computational, and cadaveric studies. Studies that did not report quantitative data under the outcomes of interest were not considered.

### 2.2. Search Strategy

This systematic review was conducted according to the Preferred Reporting Items for Systematic Reviews and Meta-Analyses: the 2020 PRISMA statement [25] and the guidelines of the Cochrane Handbook for Systematic Reviews of Interventions [26]. The PICO algorithm was preliminarily set out as:

- P (Problem): CTS;
- I (Intervention): LSIs;
- C (Comparison): CTR;
- O (Outcomes): median nerve conduction and clinical and functional outcomes.

In March 2022, the following databases were accessed: Pubmed, Web of Science, Google Scholar, and Embase. No time constrains were used for the search. The following keywords were used in combination using the Boolean operators AND/OR: carpal tunnel syndrome, median nerve entrapment, neurolysis, pain, compression, decompression, surgery, release, steroids, corticosteroids, injection.

### 2.3. Selection and Data Collection

Two authors (L.S. and F.M.) independently performed the database search. All the resulting titles were screened, and if suitable, the abstract was accessed. The full-text articles of abstracts which matched the topic were accessed. The bibliographies of the full-text articles were also screened for inclusion. Any disagreements were discussed and settled by consensus.

### 2.4. Data Items

Two authors (L.S. and F.M.) independently performed data extraction. The following data were extracted at baseline: author and year, journal, design, length of the follow-up, treatment, mean age, women as a percentage, VAS for pain (pVAS), functional (fVAS), and nocturnal paresthesia (npVAS), functional and symptom subscales of the Boston Carpal Tunnel Questionnaire (BCTQ) [27], global symptom score (GSS) [28], median nerve distal motor latency (m/s), median nerve motor amplitude (mV), median nerve sensory conduction velocity (m/s), and median nerve sensory amplitude (µV). The following data at last follow-up were extracted: npVAS, pVAS, functional and symptom subscales of the BCTQ, median nerve distal motor latency (m/s), median nerve motor amplitude (mV), median nerve sensory conduction velocity (m/s), and median nerve sensory amplitude (µV). The following complications were retrieved: major (deep infections and paresis) and minor (local pain at wrist, cellulitis, reflex sympathetic dystrophy, and wound hematoma).

### 2.5. Study Risk of Bias Assessment

The risk of bias assessment was performed by two authors (L.S. and F.M.) independently. Any disagreement was settled by a third author (N.M.). The risk of bias graph of the Review Manager Software 5.3 (The Nordic Cochrane Collaboration, Copenhagen, Denmark) was used to assess the risk of bias in the individual studies. The following biases were analyzed: selection, detection, reporting, attrition, and other sources of bias. A funnel plot of the most commonly reported outcome was used to assess the risk of publication bias. Plot asymmetries relate proportionally to the risk of publication bias.

### 2.6. Synthesis Methods

Statistical analyses were performed by the senior author (F.M.). For descriptive statistics, the IBM software version 25 was used. Mean difference (MD) and *t*-test were performed to assess baseline comparability. The meta-analyses were performed using the Review Manager Software 5.3 (The Nordic Cochrane Collaboration, Copenhagen). Binary data were evaluated through a Mantel–Haenszel analysis, with an odds ratio (OR) effect measure. The comparisons were performed with a fixed-model effect as the set up. Heterogeneity was assessed through the X^2^ and Higgins-I^2^ test. If X^2^ < 0.05 and if I^2^ test > 50%, high heterogeneity was detected. In cases of heterogeneity, a random model effect was used. The confidence intervals (CIs) were set at 95% in all comparisons. The overall effect was considered statistically significant if *p* < 0.05. Egger’s linear regression was performed through the STATA MP Software version 16 (StataCorp, College Station, USA) to assess asymmetries of the funnel plot, with values of *p* < 0.05 indicating statistically significant asymmetry.

## 3. Results

### 3.1. Study Selection

**Figure 1:**
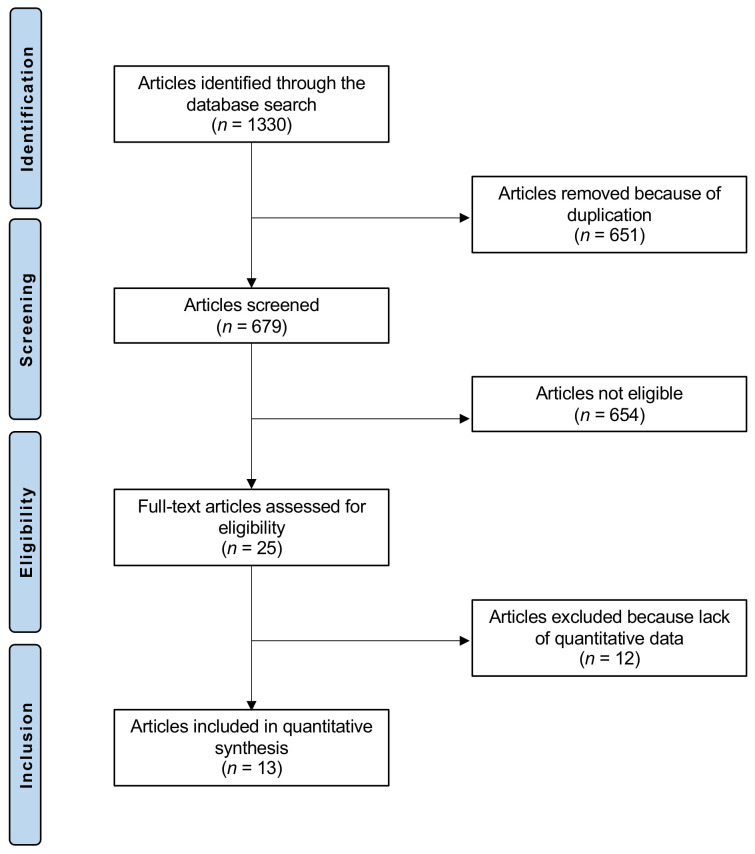
Flow chart of the literature search.

The initial literature search resulted in 1330 articles. Of these, 651 articles were removed because of redundancy, and 679 articles were screened for eligibility. Of them, 25 were comparative clinical studies which compared LSIs versus CTR. An additional 12 articles were excluded because they did not provide quantitative data on the outcomes of interest. Finally, 13 studies were included: 10 randomized, controlled trials, 1 prospective, and 2 retrospective studies. The literature search results are shown in Figure 1.

### 3.2. Risk of Publication Bias

**Figure 2:**
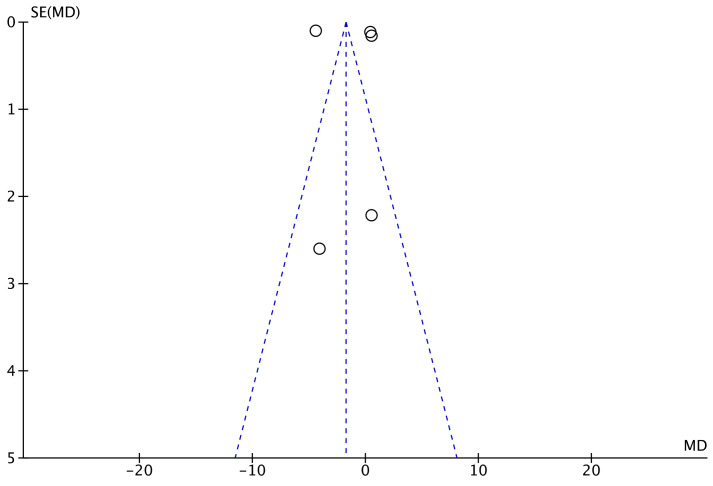
Funnel plot.

The funnel plot of the most commonly reported outcome (symptom subscale of the BCTQ) was performed to evaluate the risk of publication bias. Although some referral points were located outside the pyramidal shapes of acceptability, the plot evidenced good symmetry. To conclude, the funnel plot indicated a moderate risk of publication bias (Figure 2).

### 3.3. Study Risk of Bias Assessment

**Figure 3:**
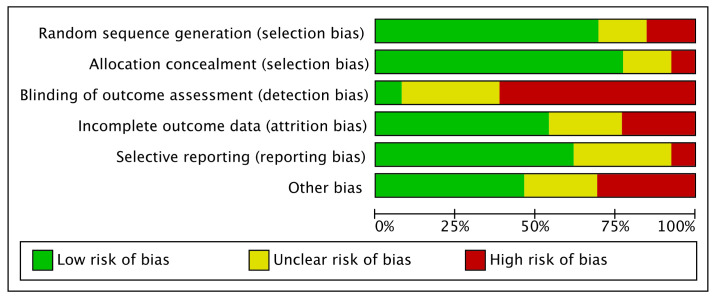
Methodological quality assessment.

The risk of bias graph demonstrated a low risk of selection bias, moderate–high risk of detection bias, and low–moderate risk of attrition and reporting biases. The risk of other bias was low–moderate. To conclude, the overall risk of bias was low to moderate (Figure 3).

### 3.4. Study Characteristics and Results of Individual Studies

Data from 1096 procedures were retrieved. The mean follow-up was 12.3 (1 to 58) months. The mean age of the patients was 51.1 ± 4.6. The generalities and baseline demographic of the included studies is shown in Table 1.

The two cohorts were found to be comparable in terms of mean age, ratio of women to men, VAS, npVAS, pVAS, fVAS, BCTQ functional and symptom subscales, GSS, distal motor latency, and sensory and motor conduction velocity and amplitude (Table 2).

### 3.5. Results of Syntheses

**Figure 4:**
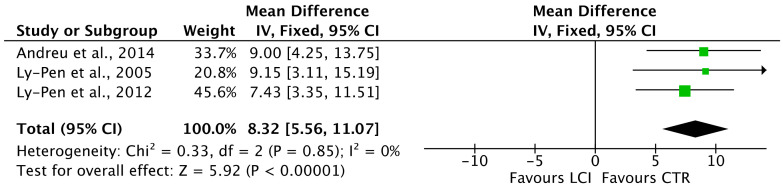
Forest plot of the comparison: npVAS.

**Figure 5:**
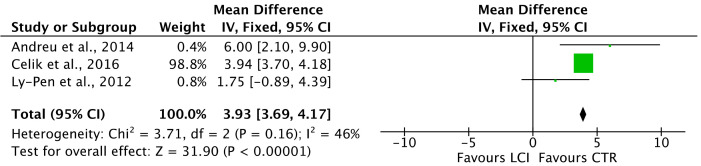
Forest plot of the comparison: pVAS.

NpVAS (MD 8.32; 95%CI 5.56, 11.07; *p* < 0.0001, Figure 4) and pVAS (MD 3.93; 95%CI 3.69, 4.17; *p* < 0.0001, Figure 5) were greater in the LSIs cohort.

No difference was found in the functional (*p* = 0.2) and symptom (*p* = 0.4) subscales of the BCTQ, median nerve distal motor latency (*p* = 0.9), median nerve motor amplitude (*p* = 0.7), median nerve sensory conduction velocity (*p* = 0.4), or median nerve sensory amplitude (*p* = 0.3).

### 3.6. Complications

Six studies reported data on complications [22,29,30,33,34,35]. No difference was found in terms of minor complications (*p* = 0.9). No major complications were observed within the duration of follow-up.

## 4. Discussion

According to the main findings of the present study, both surgical CTR and LSIs were effective and safe in reducing symptoms of CTS. CTR was associated with lower pain compared to LSIs. No difference was found in the functional and symptom subscales of the BCTQ, median nerve distal motor latency, motor amplitude, sensory conduction velocity, and sensory amplitude. Moreover, similarity was found in the rate of complications. No major complication was experienced by any patient in either cohort. Though LSIs led to greater pain relief, the current evidence shows that this superiority is not permanent. Irrespective of the severity of the symptoms, current evidence suggests that a cycle of LSIs may be considered in patients with CTS. However, patients must be aware that LSIs may be not the definitive therapy, and CTR should be recommended.

Previous evidence in the literature is conflicting. Verdugo et al. [38] conducted a systematic review on four RCTs comparing CTR versus LSIs and wrist splinting. CTR promoted better outcomes than splinting, but the superiority of LSIs was unclear [38]. Shi et al. [39] conducted a meta-analysis on seven studies. Of these, three compared LSIs versus CTR [39]. The authors found better outcomes in patients who underwent LSIs for only the first three months of treatment [39]. Klokkari et al. [19] conducted a review on 10 studies comparing conservative versus surgical management for CTS [19]. They reported better outcomes in the surgical cohort during the first 6 months, but after 12 months, no significant differences in function and symptom improvement were found [19].

LSIs have been proposed for the diagnosis of early-stage CTS [40]. The landmarks for reaching the transverse carpal ligament are identical in the included studies [20,22,23,29,30,31,33,35,36]. The needle is inserted on the ulnar side of the palmaris longus tendon proximal to the distal wrist crease [20,22,23,29,30,31,33,35,36]. Infiltration protocols differed in terms of corticosteroid type and dose. Two studies performed a second injection after two weeks if symptoms had not clinically improved [29,35,36]. Demirci et al. [31] performed a second injection after two weeks as standard.

Recent studies demonstrated the significantly superior efficacy of LSIs when compared to placebo and systemic corticosteroid administration [14,41,42]. LSIs are commonly performed for carpal tunnel syndrome with rapid clinical symptom improvement [43,44]. Serious adverse events (e.g., tendon rupture, intraneural injection, and gangrene) have an incidence of less than 0.1% [45]. Minor persistent adverse events (e.g., subcutaneous atrophy and depigmentation) were observed in 2% of patients, and transient discomfort (e.g., pain, bruising, and facial redness) was most common at 15–20% [45]. Serious complications are associated with improper technique and inadequate experience of the practitioner [43,46,47]. The majority of current studies uniformly report no significant complications or side effects and consider LSIs as safe when performed by well-trained and experienced physicians [40,43,44]. However, restitutio ad integrum cannot be guaranteed with LSIs [29]. The short-term effects of LSIs represent the main limitation of the clinical application, as up to 90% of all patients did not experience long-lasting improvement [14,17,40,41,44,48,49]. For such reasons, the use of LSIs is therefore recommended to delay surgery, during pregnancy, and in early, mild CTS for short-term purposes [40,44,49,50,51,52]. The effect of multiple injections is unclear [14,35,53,54,55]. However, there is no long-term success, even with repeated injections [35,44].

CTR represents the gold-standard treatment of severe CTS [13,18,56]. The efficacy and safety of surgical decompression in CTS is well known [15,16,17,29,57,58]. For CTR, open carpal tunnel release (ORCT) and endoscopic carpal tunnel release (ECTR) are used [15,59,60]. All studies included in the present review referred to the ORCT procedure. Ettema et al. [32] performed the endoscopic procedure in addition to the ORCT technique. Contrarily to LSIs, CTR leads to long-term success in most patients [29,39]. Nevertheless, patients must be informed that a longer period of recovery is to be expected [61]. Complications such as wound infections and tendon and/or vascular injuries are rare [62,63,64]. The most common complication is incomplete retinacular release, which is associated with symptom persistence and leads to reoperation [65,66].

The present meta-analysis certainly has limitations. Evidence from high-quality comparative clinical trials conducted on a large scale is limited. Most studies allocated patients in a random fashion [20,23,29,31,33,34,35,36]. In some studies, patients themselves decided the allocation after informed consent [21,22,30]. Two studies performed CTR in patients following unsatisfactory LSIs [32,37]. Several between-study heterogeneities were evident. Four studies included young adults [23,29,35,36]. Celik et al. [30] only considered patients aged 30-70 years of age. One study focused exclusively on the geriatric population [32]. These differences could influence the results of the present study, as work activities, regenerative capacity, and the physiological degeneration of soft tissues may differ considerably [32,67,68]. Heterogeneities in eligibility criteria were also evident. Clinical symptoms suggestive of CTS such as pain, paresthesia, hypoesthesia, tingling, burning, numbness, etc., were prerequisites in the majority of the included studies [20,22,23,29,30,31,32,33,34,35,36]. In two studies, symptoms had to be additionally confirmed by clinical tests, such as the Phalen’s, Tinel’s, and Durkan’s tests [20,34]. The duration of symptoms before surgery was also heterogeneous. Most studies required at least three months of symptom duration in order to be eligible for the intervention [29,30,33,34,35,36]. In one study, prior symptom duration was a minimum of six months [31]. Three studies did not define symptom duration as an inclusion criterion [20,21,22]. Electrophysiology of the median nerve is common for grading CTS [69,70] and was adopted by all included studies to confirm CTS [20,21,22,23,29,30,31,32,33,35,36,37]. However, this can be performed according to different methodologies and, to date, no uniformly accepted approach exists [51,69,71,72,73,74]. Electrophysiology is recommended not only for diagnosis, but also in treatment settings for CTS [75,76]. In most included studies, the severity of CTS had not been considered for patient eligibility [23,29,31,34,36,37]. Davood et al. [20] focused on patients with mild to moderate CTS according to Bland’s criteria [71]. Two studies [21,31] only considered moderate CTS according to the classification of Visser [51] or the Padua criteria [73]. Ettema et al. [32] included mild to severe CTS according to AAEM criteria [74]. Gurcay et al. [22] only included severe CTS according to Shin [77]. Moreover, given the lack of available quantitative data, studies were included regardless of the type of steroids used for the treatment (e.g., betamethasone or prednisolone). Some authors combined LSIs with local anesthetics (e.g., lidocaine); however, given the lack of quantitative data and information, it was not possible to investigate whether the addition of local anesthetics may influence the outcome. Given these limitations, results from the present study must be interpreted with caution. The current literature would benefit from high-quality clinical trials on a large scale.

## 5. Conclusions

Both CTR and LSIs were effective and feasible in reducing symptoms of carpal tunnel syndrome. Though LSIs led to greater pain relief, this superiority was not permanent. Irrespective of the severity of the symptoms, current evidence suggests that a cycle of LSIs may be considered in patients with CTS. However, patients must be aware that LSIs may be not the definitive therapy, and CTR should be recommended. These results should be considered within the limitations of the present meta-analysis.

## Figures and Tables

**Table 1:** Generalities and patient baselines of the included studies (LSIs: local steroid injections; CTR: carpal tunnel release).

Author, Year	Journal	Design	Follow-up (Months)	Treatment	Procedures (*n*)	Mean Age	Women (*%*)
Andreu et al., 2014 [29]	*Clin Neurophysiol*	Randomized	12	LSIs	83	53.0	92
				CTR	80	50.0	
Celik et al., 2016 [30]	*J Clin Neurophysiol*	Prospective	6	LSIs	50	50.8	84
				CTR	50	51.4	94
Davood et al., 2018 [20]	*Shafa Orthop J*	Randomized	6	LSIs	33	45.4	81
				CTR	35	46.2	82
Demirci et al., 2002 [31]	*Rheumatol Int*	Randomized	6	LSIs	46	45.3	91
				CTR	44	48.0	86
Ettema et al., 2006 [32]	*Plast Reconstr Surg*	Retrospective	58	LSIs	41		
				CTR	47		
Güvenç et al., 2019 [21]	*Eur Res J*	Retrospective	6	LSIs	33	51.0	81
				CTR	78	53.5	91
Gurcay et al., 2017 [22]	*Turk Neurosurg*	Randomized	1	LSIs	21	61.4	94
				CTR	18	56.8	88
Hui et al., 2005 [33]	*Neurology*	Randomized	5	LSIs	25	48.2	96
				CTR	25	50.8	96
Ismatullah et al., 2013 [34]	*JPMI*	Randomized	3	LSIs	20	46.9	70
				CTR	20	43.8	75
Ly-Pen et al., 2005 [35]	*Arthritis Rheum*	Randomized	12	LSIs	49	53.2	92
				CTR	56	50.5	
Ly-Pen et al., 2012 [36]	*Rheumatology*	Randomized	24	LSIs	49	50.0	92
				CTR	56	53.0	
Seror et al., 1992 [37]	*J Hand Surg Br*	Randomized	9	LSIs	56	58.6	81
			12	CTR	33	57.5	76
Wheab et al., 2019 [23]	*Res J Pharm Tech*	Randomized	12	LSIs	24		
				CTR	24		

**Table 2:** Baseline comparability (LSIs: local steroid injections; CTR: carpal tunnel release; pVAS: VAS for pain; fVAS: functional VAS; npVAS: nocturnal paresthesia VAS; BCTQ: Boston Carpal Tunnel Questionnaire; GSS: global symptom score).

Endpoint	LSIs (*n* = 530)	CTR (*n* = 566)	MD	*p*
Mean age	51.3 ± 5.1	51.0 ± 4.1	0.2	0.9
Women (%)	85.8 ± 8.3	86.8 ± 7.5	−1.0	0.8
VAS	31.1 ± 38.3	30.7 ± 37.9	0.4	1.0
npVAS	58.0 ± 0.1	55.5 ± 0.5	2.5	0.07
pVAS	42.1 ± 0.2	42.2 ± 0.4	−0.1	0.7
fVAS	38.0 ± 0.1	39.0 ± 0.1	−1.0	0.09
BCTQ functional	8.4 ± 11.1	7.8 ± 9.6	0.6	0.9
BCTQ symptom	10.7 ± 15.2	11.0 ± 15.7	−0.3	1.0
GSS	30.0 ± 6.8	32.0 ± 4.8	−2.0	0.8
Distal motor latency (m/s)	5.3 ± 0.6	5.9 ± 1.0	−0.6	0.4
Motor amplitude (mV)	5.1 ± 2.1	5.1 ± 2.2	0.0	1.0
Motor conduction velocity (m/s)	45.8 ± 8.8	42.8 ± 0.8	3.0	0.7
Sensory conduction velocity (m/s)	32.3 ± 10.3	28.4 ± 10.6	3.9	0.6
Sensory amplitude (µV)	14.2 ± 10.9	11.0 ± 6.8	3.3	0.7

## Data Availability

The datasets generated during and/or analyzed during the current study are available throughout the manuscript.
